# Personalizing Approaches in International Projects Engaging Individuals with Vulnerabilities: The Lessons Learned for a Person-Centered Research

**DOI:** 10.3390/jpm16060296

**Published:** 2026-05-31

**Authors:** Stefania Chiappinotto, Chiara Moreal, Aysun Bayram, Nevenka Kregar Velikonja, Aysel Özsaban, Montserrat Solà-Pola, Alba Roselló-Novella, Kinga Zdunek, Beata Dobrowolska, Alvisa Palese

**Affiliations:** 1 Department of Medicine, University of Udine, 33100 Udine, Italy; stefania.chiappinotto@uniud.it (S.C.); moreal.chiara@spes.uniud.it (C.M.); 2 Department of Fundamentals Nursing, Karadeniz Technical University, Trabzon 61080, Türkiye; aysunbayram@ktu.edu.tr (A.B.); ayselozsaban@ktu.edu.tr (A.Ö.); 3 Faculty of Health Sciences, University of Novo Mesto, 8000 Novo Mesto, Slovenia; nevenka.kregar-velikonja@uni-nm.si; 4 Faculty of Nursing, University of Barcelona, 08907 Barcelona, Spain; montsesolap@ub.edu (M.S.-P.); albarosello@ub.edu (A.R.-N.); 5 Health Education Department, Medical University of Lublin, 20-059 Lublin, Poland; kinga.zdunek@umlub.edu.pl; 6 Department of Holistic Care and Management in Nursing, Medical University of Lublin, 20-059 Lublin, Poland; beata.dobrowolska@umlub.edu.pl

**Keywords:** ethics, multi-country studies, person-centered, research, vulnerability

## Abstract

**Background:** The involvement of people living in situations of vulnerability has long been a central ethical issue in research, particularly in contexts marked by power asymmetries, limited access to resources, or restricted decisional autonomy. Although international ethical frameworks offer increasing guidance on protecting vulnerable participants, applying these principles in everyday research practice remains challenging, especially in qualitative and multi-country studies. This communication draws on the experience of the European Protecting You & Others project, an Erasmus+ initiative conducted across five countries. **Methods:** During the project design and implementation, the research team engaged in ongoing reflexive work to examine the ethical, methodological, and practical challenges encountered when defining vulnerability, involving participants living in situations of vulnerability, and adapting research activities and educational interventions to different specific needs. Reflexive notes and collective team discussions were used to identify recurrent challenges and the strategies adopted to address them. **Results:** Key challenges included (a) the difficulty of choosing an inclusive yet operational definition of vulnerability; (b) participants’ self-perceptions and tensions between externally assigned vulnerability; (c) risks of stigmatization associated with categorization; the use of respectful and context-appropriate language; and (d) the adoption of a shared international framework adapted to educational content across countries. Overall, vulnerability emerged as a dynamic and context-dependent condition that requires research designs, methodologies and interventions to remain open, flexible, and responsive throughout the study process. **Conclusions:** Studies involving people living in situations of vulnerability, particularly in international and multi-country contexts, should not rely solely on predefined classifications or standardized safeguards. Instead, adaptive procedures are needed to recognize how needs, barriers, resources, and forms of participation vary across individuals and contexts. Such openness may support more person-centered approaches to engagement, communication, and intervention adaptation, while preserving ethical consistency and methodological rigor across countries.

## 1. Introduction

The involvement of people living in situations of vulnerability has long been a central ethical issue in human research. The need to protect individuals who may be exposed to coercion, exploitation, or impaired decisional freedom was first articulated internationally in the Nuremberg Code [1], which established voluntary consent as a fundamental condition for research. This ethical trajectory was later expanded through key international documents, including the Declaration of Helsinki of the World Medical Association, first adopted in 1964 [2]; the International Ethical Guidelines for Health-related Research Involving Humans issued by the Council for International Organizations of Medical Sciences, first developed in 1982 [3]; the Convention on Human Rights and Biomedicine of the Council of Europe [4]; the United Nations Educational, Scientific and Cultural Organization (UNESCO) Universal Declaration on Bioethics and Human Rights [5]; and the Guideline for Good Clinical Practice issued by the International Council for Harmonization of Technical Requirements for Pharmaceuticals for Human Use, most recently revised in 2025 [6].

Protecting participants in situations of vulnerability is even more complex and is fundamentally recognized in all research ethics frameworks [7]. International debate has increasingly focused not only on how people in situations of vulnerability should be protected, but also on how vulnerability itself should be defined, identified, and operationalized in research practice. A first major challenge concerns the theoretical approach to vulnerability, specifically how vulnerability is defined, who is considered vulnerable, and on what basis [7]. A second, more practice-oriented challenge concerns how vulnerability is addressed in research processes, including, for example, participant involvement, gatekeeping, and interview dynamics.

Despite increasingly conceptual and normative rules on vulnerability, translating these principles into everyday research practice remains an issue [8,9]. Safeguards may become procedural and compliance-driven, with consent and “extra protections” linked to the status of vulnerability reduced to standardized checklists that do not fully capture situational changes in power, dependence, or distress during fieldwork [10]. These issues are particularly evident in qualitative designs, where emergent data collection dynamics can generate unanticipated risks that are not easily managed by pre-specified procedures [10]. In these study designs, participation may involve the disclosure of sensitive experiences and increased relational proximity between researcher and participant, raising specific ethical challenges for individuals and groups in situations of vulnerability [11]. Furthermore, although ethical frameworks emphasize tailored safeguards, multi-country research often faces additional issues due to different rules and implementation challenges, as context-sensitive intentions are difficult to operationalize consistently across sites [12].

Overall, these issues are relevant to the broader debate on personalized and precision-medicine approaches in healthcare and research. While precision medicine is mainly associated with biological, genomic, or clinical stratification, personalization in research with people living in situations of vulnerability requires attention to contextual, relational, communicative, and social determinants that influence participation, access, and benefit. From this perspective, personalizing research procedures means adapting engagement, consent procedures, communication strategies, educational materials, and intervention delivery to participants’ specific needs.

The “Protecting You & Others” (PRO Y&O) project is a European initiative aimed at strengthening preventive behaviors for respiratory infectious diseases among people living in vulnerable situations, as well as training healthcare and community stakeholders to promote sustainable implementation of educational strategies through community resources and public health nursing. During project development and the involvement of people experiencing various vulnerabilities, the research team encountered substantial challenges. These challenges influenced the project’s design and implementation choices and provided the rationale for this discussion paper on how vulnerability should be negotiated in practice within multi-country, multi-stakeholder research. Therefore, the aim was to describe challenges encountered by the PRO Y&O team in addressing ethical issues and personalizing interventions involving individuals living in situations of vulnerability, with attention to how person-centered and context-sensitive approaches can inform research practice, and to discuss how these challenges may be addressed.

## 2. Materials and Methods

This discussion paper emerged from debates during the activities of the PRO Y&O project as an international Erasmus+ project involving five countries, which began in 2024 (Table 1). Its main aims were to develop an inclusive, community-based infection prevention behavior change program for people living in various situations of vulnerability, integrating digital and health literacy principles. In this project, personalization was understood as the adaptation of educational and research procedures to specific needs, barriers, resources, and contexts. Development included:

(1) evidence synthesis through literature reviews on behavior change theory and techniques, as well as on educational interventions designed to foster self-care behaviors and prevent respiratory infections among people with vulnerabilities;
(2) a qualitative analysis regarding educational needs and preferred educational strategies as perceived by populations in vulnerable situations and their caregivers to inform the program framework; and
(3) the creation and validation of an assessment tool for infection-prevention-related behaviors; and a multilingual Massive Open Online Course (MOOC) on prevention of respiratory infections in vulnerable populations for carers working with vulnerable individuals. Course materials were produced in English and translated into Italian, Polish, Slovenian, Spanish, and Turkish, with selected audio-visual content additionally adapted into the sign language of each national language as well as English sign language. These adaptations were intended to increase linguistic, sensory, and cultural accessibility.

Throughout the various project phases, over 100 people in vulnerable situations and more than 50 carers were involved to gather their educational needs, preferences and validate the MOOC and related tools developed by the consortium.

During the project, the research team adopted a structured reflection-in-action process [13,14], particularly in the consortium meetings held every two months, where ethical, methodological, and practical issues were discussed as they arose. To support this process, two researchers (SC, CM) maintained a reflective journal [15] to document the critical issues raised, the different interpretations expressed by consortium members, the alternatives considered, and the practical decisions made. This phase aimed to describe and critically organize the main challenges encountered across the various project activities. The reflective material was structured according to the following elements: (a) the problem raised; (b) the contents of the discussion among consortium members, including convergences, divergences, and possible alternatives considered in light of the available literature; (c) the reasons for following or not following evidence-based directions; (d) the decisions made; and (e) the practical rationale for the choice.

The synthesized reflective summaries were then analyzed through an iterative descriptive synthesis. They were then shared with all members of the research team (see authors) for review, consensus, and further comments or clarifications. Through this iterative process, five main issues addressed by the consortium were identified, namely: (1) defining vulnerability, a difficult choice; (2) being or feeling vulnerable; (3) classifying vulnerability: protection or stigmatization; (4) use of an appropriate language: common words or personalized terms; and (5) applying a shared international framework: toward a care based on the context.

For each issue, the following result section presents the key challenge identified by the consortium, examples emerging from the PRO Y&O project, the decisions made during project implementation, and the main lesson learned.

## 3. Results

### 3.1. Defining Vulnerability, a Difficult Choice

From the earliest stages of the project, it became clear that the consortium needed to reach a shared understanding of the concept of vulnerability and its various expressions or forms to support the subsequent personalization of the educational intervention. This immediately raised a major methodological issue for the research team, as the entire protocol design depended on a shared concept of vulnerability.

Vulnerability is inherently complex to define. According to the ethics of care, vulnerability is considered an anthropological characteristic of human beings [16,17]. This approach emphasizes that vulnerability is not limited to age or specific life conditions; rather, it is intrinsic to the relational nature of human existence [18]. Recent studies confirm that no single, universally accepted definition exists and that the concept continues to evolve across ethical, legal, and empirical debates [18,19]. In general, the literature commonly describes vulnerability as an increased likelihood of being harmed, wronged, or inadequately able to protect one’s own interests within a given research context [20,21]. The Medical Subject Headings term “Vulnerable Populations” in PubMed platform refers to groups whose range of options is severely limited, who are frequently subject to coercion in decision-making, or whose capacity to provide informed consent may be compromised. At the same time, a growing body of literature has challenged approaches that treat vulnerability as a fixed characteristic of predefined groups. Instead, contemporary papers increasingly support relational, situational, and dynamic interpretations that better capture how vulnerability emerges through context, dependency, power asymmetries, and institutional arrangements [8,10,19].

Within our project, the concept of vulnerability needed to be broad and inclusive enough to encompass the heterogeneity of populations potentially targeted by the intervention, while remaining sufficiently operational to guide the design of tailored educational strategies. In practical terms, this meant that the consortium could not select target groups only on the basis of broad demographic labels. In the field of infection prevention and control, this challenge is particularly evident, as vulnerability may arise from multiple and overlapping factors. People with pre-existing health conditions may be more exposed to severe consequences of infection; children and older adults may be at increased risk due to age-related physiological susceptibility, dependence, or limited autonomy; and individuals facing social disadvantage may encounter structural barriers that make preventive behaviors difficult to access. For example, people experiencing homelessness or severe economic deprivation may have limited access to water, hygiene facilities, or health services, reducing their ability to adopt even basic infection-prevention measures. Similarly, people who use drugs or experience strong social stigma may face additional barriers related to unstable living conditions, marginalization, or reduced engagement with health-promoting behaviors. Other sources of vulnerability stem from difficulties in accessing or processing educational content itself. People with visual impairments may not be able to access written material, deaf individuals may not benefit from audio-based resources, and migrants or mobile populations may not fully understand content delivered in a language different from their own. Likewise, people with low health literacy may also be considered vulnerable, as they often have difficulty understanding and using health information, which can affect their ability to manage their own health and navigate healthcare systems appropriately. This situation is frequently linked to a limited understanding of how health services operate in their context. As a result, these individuals tend to show lower self-care capacity, poorer adherence to preventive and therapeutic recommendations, and less efficient use of health and social services, including repeated consultations and avoidable emergency visits [22,23].

Vulnerability does not arise solely from individual characteristics or health conditions; it is also influenced by the social, economic, and institutional circumstances in which individuals live. Factors such as poverty, low educational attainment, unemployment, insecure housing, limited access to healthcare, discrimination, social exclusion, and digital inequality can significantly impede individuals’ ability to adopt infection prevention behaviors and access health information. Therefore, vulnerability should be assessed not as an inherent attribute of individuals, but as a condition shaped by the interaction between lived circumstances, available resources, environmental constraints, and structural determinants. This perspective aligns with the Social Determinants of Health framework, which emphasizes that health outcomes are shaped not only by biological factors but also by the conditions in which individuals are born, raised, live, and work [24].

Another important point is that vulnerability is not always a fixed or permanent condition. Some forms of vulnerability may be chronic and long-term; for example, severe disability, cognitive decline, or persistent poverty may increase individuals’ need for ongoing support. Conversely, some vulnerabilities are temporary and situational; events such as job loss, acute illness, migration, natural disasters, or pandemics may make individuals more vulnerable during specific periods. This distinction is important in designing educational interventions, as persistent vulnerabilities require more sustained and tailored support mechanisms, while temporary vulnerabilities may require strategies that are more flexible and capable of providing rapid responses.

Overall, identifying a single definition of vulnerability that encompasses these different expressions proved far from straightforward. The PRO Y&O consortium therefore faced a central tension in international vulnerability research: how to adopt a concept broad enough to recognize heterogeneous forms of disadvantage across countries, while keeping it sufficiently operational to guide the identification of target groups and the personalization of educational interventions. For this reason, vulnerability was not treated as a fixed label attached to predefined groups, but as a context-dependent condition to be interpreted in relation to barriers, resources, and participation needs. This approach allowed the consortium to maintain a shared conceptual orientation across countries while allowing for local adaptation in the selection of populations and the design of tailored educational strategies.

We learned that in international projects, vulnerability should first be defined as a shared concept and then operationalized locally according to the barriers, resources, and educational needs identified in each setting, as a prerequisite for meaningful personalization of research and educational interventions.

### 3.2. Being or Feeling Vulnerable

A further concern arose when PRO Y&O fieldwork began. In practice, vulnerability did not appear as a stable “condition”. Instead, it often emerged as a contextual, situational, and partly subjective state. For example, in some meetings with people experiencing homelessness, some individuals refused to be interviewed not because they objected to the interview itself, but because they did not perceive themselves as having particular health needs or as living in a vulnerable situation. In other cases, individuals considered vulnerable by definition due to chronic illness were very active and engaged in social life and did not consider themselves vulnerable. Thus, the issue of subjective and objective vulnerability arose.

Similarly, researchers have recognized that vulnerability is linked to life circumstances. For example, when the research team included older people living in an isolated mountain town with low digital literacy among potentially vulnerable populations, this classification initially appeared justified. They were geographically isolated, had limited access to services, and could not easily obtain health-related information through digital channels. However, during the COVID-19 pandemic, that same spatial isolation also functioned as a form of protection, reducing exposure to infection. Thus, a characteristic initially interpreted as indicating vulnerability became, in that specific context, a relative source of advantage. When interview questions focused specifically on vulnerability during the pandemic, this paradox became evident and challenged the internal consistency of the initial categorization.

The literature similarly suggests that, in some cases, individuals generally considered vulnerable may perceive themselves as only minimally vulnerable, or not vulnerable at all, and this perceived invulnerability may itself increase risk [25]. Furthermore, some individuals living in objectively vulnerable circumstances may not perceive themselves as vulnerable because they have strong social networks, prior life experiences, or personal coping strategies. This demonstrates that vulnerability should also be assessed in terms of individuals’ existing resources, resilience, and capacity to adapt to adverse conditions [26]. This tension is also reflected in processes of self-perception and self-identification. For example, studies involving people within the autism spectrum have shown that preferred forms of self-description may vary and may, in some cases, reflect autism as part of personal identity. This highlights how external categorizations may not fully coincide with how individuals perceive and define themselves [27].

Overall, the experience of the PRO Y&O project highlighted a core tension in international vulnerability research: whether vulnerability should be understood primarily as an attribute assigned by researchers on the basis of predefined criteria, or as a condition that must also be interpreted through participants’ own perceptions, lived experiences, and specific cultural contexts. The discrepancy between being classified as vulnerable and feeling vulnerable may substantially influence participants’ willingness to engage in research, the relevance of the proposed intervention, and the ethical legitimacy of the categories used by researchers.

We learned that it is important to avoid assuming that externally assigned vulnerability automatically corresponds to participants’ self-perception, and to consider perceived needs, available resources, and willingness to engage as part of the recruitment and intervention adaptation process.

### 3.3. Classifying Vulnerability: Protection or Stigmatization

The previous reasoning led to another challenge faced by the consortium: identifying a suitable classification of vulnerability. The literature increasingly recognizes that some form of classification is often necessary to translate ethical principles into operational decisions; however, any attempt to classify vulnerability must also confront the risk of stigmatizing, victimizing, or oversimplifying the individuals such classifications are intended to protect [28,29]. In this sense, classification is both necessary and ethically problematic. Such distinctions may be analytically useful in translating ethical principles into practice, as they can support the design of proportionate, non-paternalistic protections that uphold autonomy while recognizing dependence and unequal power relations, and help prevent research procedures themselves from becoming sources of harm or “pathogenic” vulnerability [8,30].

There are numerous categorizations of vulnerability. For example, some authors have proposed a broad theoretical distinction between vulnerability grounded in ontological foundations, which highlights the existential condition of human finitude and dependence, and vulnerability grounded in circumstantial foundations, which refers to social and individual structures such as culture, social position, or life conditions that may generate or intensify vulnerability [8]. However, although conceptually robust, this distinction did not adequately support the practical needs of our project, as it did not help identify the specific needs that differentiated participants in vulnerable situations from others, nor did it offer sufficiently concrete guidance for developing an educational intervention responsive to different vulnerability profiles. Alternative classifications have also been proposed. For example, Schrems [29] distinguishes between consent-based vulnerability, which relates to decisional capacity and the informed consent process; harm-based vulnerability, which concerns an increased risk of harm due to physical, mental, or social predispositions; and global vulnerability, which combines both dimensions. This framework was helpful in clarifying the ethical mechanisms through which vulnerability may arise, but it was less useful for identifying project-specific target groups and for guiding the concrete personalization of educational content. In our case, these categories do not help to identify the practical factors that made a person vulnerable in relation to infection prevention and health education in ways that could directly inform intervention design.

After several meetings and discussions, the PRO Y&O research team agreed that Gordon’s classification of vulnerability [31] was the most suitable to support the project, as it organizes different forms of vulnerability into practical categories that can be translated into intervention design, rather than merely discussing vulnerability at an abstract conceptual level. This framework distinguishes between types of vulnerability based on people’s situations: cognitive or communicative vulnerability, referring to individuals who may have difficulty understanding information and making decisions about participation; institutional vulnerability, including people under the formal authority of others who may hold different values, goals, and priorities from the potential participant; deferential vulnerability, concerning those under the informal authority of others; medical vulnerability, including people with serious health conditions and limited treatment options; economic vulnerability, referring to individuals disadvantaged in the distribution of social goods and services; and social vulnerability, including those belonging to socially undervalued groups [32]. For the purposes of PRO Y&O, this classification was particularly useful because it provided sufficiently differentiated categories to guide the identification of target populations and the tailoring of educational strategies. It allowed the consortium to link specific forms of vulnerability with practical adaptations, for example, by combining written and audio-visual materials for people with sensory impairments or by simplifying content for those with limited health or digital literacy. Although this classification offers significant practical advantages, categorizing vulnerability inevitably carries certain risks of labeling and stigmatization. Therefore, it is important to apply each category in a context-sensitive and careful manner.

However, adopting this framework raised another problem: which formal criteria, at least in the research protocol, should be used to determine whether a person or group should be considered vulnerable within each category? A practical example concerns social vulnerability. Within the project, this category included Roma communities. This immediately raised an ethical and methodological question: is belonging to an ethnic minority group, in itself, sufficient to justify classification as vulnerable, or should such classification depend on additional contextual factors and lived conditions? In discussing this issue within the international research team, it became clear that belonging to an ethnic minority group or a migrant community, whether settled or mobile, may be associated with reduced access to health services, lower familiarity with the local language, limited interaction with majority populations, different cultural beliefs and practices, and greater exposure to social exclusion or discrimination. Yet these factors cannot be presumed to apply uniformly to all individuals within that group [33]. Therefore, the risk of classification is that a category introduced to facilitate protection may inadvertently essentialize identities and attribute vulnerability where it is only partial, situational, or not experienced as such by participants themselves.

A recurring recommendation in the literature is to tailor safeguards to the specific sources and expressions of vulnerability that arise within a given study, rather than applying uniform protective measures based solely on categorical labels [11,32]. From this perspective, classifications can serve as orientation tools, but they should not be regarded as definitive ethical conclusions. Instead, they need to remain open to contextual refinement, participant perspectives, and ongoing negotiation throughout the research process. For example, in studies involving fluctuating decision-making capacity, such as with individuals with dementia, process consent operationalizes autonomy as a continuous and interactional practice through repeated checks of assent and willingness before, during, and after each research encounter, with clear stopping rules when discomfort or dissent emerges [34]. Similarly, participants may resist externally assigned categories of vulnerability and instead describe their condition as variable, contextual, and closely intertwined with dignity and autonomy, requiring researchers to calibrate protections without imposing paternalistic interpretations [35]. For example, the ways of describing autism are not uniformly shared across groups: autistic people may prefer forms of self-description that do not coincide with those endorsed by professionals, highlighting a possible misalignment between external categorization and individuals’ own understanding of themselves [36].

With the PRO Y&O project, researchers learned that it is not enough to simply classify vulnerability. However, using classification as a flexible and revisable framework can guide the personalization of educational strategies without reducing participants to predefined categories or obscuring the contextual conditions that shape their vulnerability.

The lesson learned was that classifications can support involvement but should be considered as provisional orientation tools rather than fixed labels to apply.

### 3.4. Use of an Appropriate Language: Common Words or Personalized Terms?

After conducting preliminary research to inform the personalization of the educational intervention, including the development and piloting of the MOOC, we proceeded to write scientific articles to communicate. At this stage, an additional issue emerged concerning the language used in this context. Our approach was to use common, respectful, and ethical language, but one that was standardized across populations.

However, the response after the first round of peer review was swift. The reviewers emphasized that guidelines exist in the literature regarding appropriate language use. In particular, the Centers for Disease Control and Prevention (CDC) outlines guiding principles to promote truly inclusive communication in public health, emphasizing that language is an essential tool for advancing equity. Using respectful, non-stigmatizing, and people-centered language enables researchers to address disparities without blaming individuals or communities, acknowledging that many vulnerabilities result from deep-rooted social and systemic inequalities. Applying a “health equity lens” means evaluating the impact of messages, building on community strengths, recognizing the intersectionality of identities, and developing programs and recommendations through dialog that builds trust and encourages shared participation [37].

Moreover, a document from the National Institutes of Health on inclusive language explains how to choose terms that respect individuals’ dignity and prevent stigma. It promotes person-first language (for example, “people with a disability” rather than “disabled”), while acknowledging that some communities prefer identity-first language, such as the deaf and autistic communities. The guidance covers numerous areas—from disability to mental health, substance use to housing, immigration to older age, poverty to gender identity—suggesting ways to describe conditions and contexts without attributing blame, dehumanizing, or oversimplifying complex groups. For example, it recommends “people who use drugs” instead of “drug users”, “people living with HIV” instead of “HIV-positive”, “people experiencing homelessness” rather than “the homeless”, and “immigrants” rather than “illegal aliens”. The document also highlights the importance of engaging with the communities involved to understand their preferred terms, avoiding words with violent or instrumental connotations such as “target” when referring to people, and using collective descriptions with care.

This was particularly relevant to the PRO Y&O project, where some populations needed to be identified at the group level to guide recruitment, adaptation, and intervention planning. In this context, the key issue was not to avoid group terms entirely, but to use them precisely, respectfully, and in a contextually grounded manner, without implying that vulnerability applies uniformly to all individuals within a given group. It also emphasizes that language evolves and that effective communication depends on listening, cultural awareness, and the intention to represent people accurately and respectfully [38].

Within the PRO Y&O project, this issue translated into a tension between linguistic personalization and cross-contextual consistency. On one hand, researchers need a deep understanding of the community or group, however small, with which they work or conduct research, to use personalized language that respects their wishes and needs. On the other hand, there is an intention to include as many people as possible in educational opportunities to expand potential benefits, recognizing that the language used may not be appropriate for all populations in situations of vulnerability and may violate some of the ethical principles inherent to their condition. Specifically, efforts to avoid stigma have raised questions about how vulnerability should be defined and communicated in ways that remain respectful and acceptable to participants, while still allowing the intervention to be operationalized consistently. In parallel, translating inclusivity commitments into practice has required concrete decisions on accessibility and equity: how to ensure that the program is truly usable and accessible for people with diverse vulnerabilities, and how to balance comparability across contexts with the need for local adaptation.

Implementation science and adaptation studies note that translating interventions into diverse contexts requires more than simple linguistic translation: cultural and contextual adaptation is iterative, demands significant resources, and can fail when responsibilities, stakeholder priorities, and local capacities diverge, creating uncertainty about how best to implement adaptation practices in the field [39,40]. Linguistic and cultural adaptation in highly diverse contexts further highlights that, beyond translation challenges, factors such as trust, relevance, and ongoing engagement can limit scalability in real-world settings, even when effectiveness is demonstrated in controlled environments [41]. On such a sensitive topic as vulnerability and the implementation of preventive behaviors after a highly controversial pandemic, the group reflected deeply on the words to use, the tests to conduct, and how to avoid situations that could be stigmatizing or offensive.

Moreover, in research contexts, many contributions have moved beyond identifying “vulnerable groups” to propose context-sensitive ethical strategies aimed at reducing risks without treating vulnerability as a fixed attribute of populations with particular characteristics [10,32]. In parallel, critiques of ethics governance highlight that overly standardized, rule-based protections, particularly regarding informed consent, may become routinized and poorly aligned with qualitative, emergent designs, reinforcing the need for flexible, context-sensitive safeguards rather than uniform procedures [10]. Recent nursing ethics scholarship also cautions that protective responses grounded in categorical vulnerability attributions may become disproportionate and themselves generate harm, while obscuring structural and environmental determinants of risk, further supporting flexible, context-sensitive safeguards [30]. At the same time, work on global research governance highlights that multi-site projects can be constrained by varying ethics committee expectations and procedural requirements, which may push teams toward uniform documentation rather than adaptive, relational safeguarding [10]. It also remains an open question whether attributing vulnerability to a person stigmatizes them [30], as they are included specifically because of their disadvantageous characteristics in everyday life, which may establish a position of power over them. This shift in language acknowledges that framing vulnerability in this way helps to avoid stigmatization and recognizes the dynamic and situational nature of exposure to risk.

A further ethical and methodological challenge was ensuring direct and equitable communication with all groups involved. Communicative, linguistic, or cognitive differences sometimes limited participants’ direct engagement, requiring adaptations to recruitment, data collection, and educational activities. For example, during data collection with autistic individuals, communication and cognitive differences made it difficult to conduct interviews in a manner that was both effective and ethically appropriate for the participants. Consequently, in some cases, the consortium decided to interview caregivers rather than individual with vulnerabilities. This occurred when direct interviewing risked becoming excessively burdensome or ineffective, for example when participants had difficulties sustaining a conventional interview, understanding abstract questions, or expressing preferences about educational needs and infection-prevention behaviors. In these cases, caregivers were asked to reconstruct everyday barriers, communication preferences, feasible educational formats, and adaptations needed to make the MOOC materials more accessible. For example, participants with hearing impairments, participation was limited in situations where sign language support could not be provided or alternative communication tools were insufficient. Similarly, establishing effective communication with migrant participants who do not have sufficient proficiency in the research language (the national language or English) raised additional ethical concerns due to the necessity of language mediation. This decision was not intended to replace participants’ voices, but to preserve ethical proportionality when direct participation was not feasible or could compromise comfort, dignity, or meaningful engagement.

The literature suggests adapting all educational material produced for this population [41,42]; this was, in fact, the approach taken by the researchers of the PRO Y&O project. However, concrete experience demonstrates that the principle of inclusivity in research is not always fully realized and that access to certain vulnerable groups can be limited due to structural and communicative barriers. Therefore, we learned that such limitations should be regarded not merely as a methodological constraint but also as a significant area of tension directly related to the principles of equal participation and representation in research ethics.

### 3.5. Applying a Shared International Conceptual Structure: Toward a Care Based on the Context

A distinct challenge arose when the consortium attempted to apply a shared vulnerability conceptual structure across different national contexts. Although Gordon’s classification [31] provided a common conceptual structure for selecting target populations, its practical application varied by country. Categories that appeared coherent at the project level did not always retain the same relevance, visibility, or ethical acceptability within each local context.

First, a category could be highly relevant in one country but less so in another. For example, geographical isolation may pose a major barrier to prevention where remote communities have limited access to primary care, public health services, or educational initiatives, but may be less significant in contexts where territorial services are more evenly distributed or where digital and community-based resources mitigate the effects of distance. Second, a category could be visible in one setting but less visible in another. For instance, informal caregivers or people providing care within the family may be easily identifiable where care responsibilities are formally recognized by services or community organizations, but remain less visible in countries where family care is largely private, undocumented, or not explicitly connected to health-promotion pathways. Third, a category could be ethically acceptable in one context but more problematic in another. For example, identifying migrants, ethnic minorities, or Roma communities as target groups may be considered appropriate where there are well-documented barriers to accessing health information and services, but it may also risk reinforcing stigma or reducing heterogeneous experiences to a single group label if local histories of discrimination, political sensitivity, or community preferences are not carefully considered [10,19].

This asymmetry became evident during the national selection of target populations. While all partners operated within the same overall conceptual structure, each country identified populations that reflected its own social, territorial, and institutional realities, as well as its healthcare system. As a result, the chosen groups were locally meaningful but not equivalent across sites. For example, a group selected because of limited digital literacy in one country could not be considered equivalent to a group selected because of linguistic or migration-related barriers in another country, even though both were included under the broader vulnerability conceptual structure. The practical decision was therefore to document the local rationale for each population selected, rather than assuming that the same category had the same meaning across countries.

This raised a methodological question that went beyond adaptation: what exactly was being compared across countries? If two populations were both included under a broad label of vulnerability, this did not necessarily mean they experienced the same type, intensity, or mechanisms of disadvantage, nor that they faced comparable barriers to educational access or preventive action. The shared classificatory language thus created a degree of formal consistency, but not full analytical equivalence [10,43].

From this perspective, the multi-country design revealed a significant tension between coordination and comparability of results. On one hand, using a common conceptual structure was necessary to design the project, justify group selection, and maintain coherence across partners. On the other hand, local application of that conceptual structure depended on micro-contextual conditions, including welfare arrangements, service availability, territorial isolation, patterns of marginalization, and culturally specific relationships with institutions. Consequently, the same group could correspond to different empirical realities in different countries. What constituted a major source of vulnerability in one setting could be secondary, mitigated, or differently expressed in another. This also complicated the interpretation of findings. Including multiple countries might initially suggest broader applicability and stronger generalizability of findings. However, the PRO Y&O experience indicated that this assumption is less straightforward than often implied. Cross-national diversity increased heterogeneity and made comparison more difficult. The wider the range of contexts included, the greater the possibility that the selected groups would reflect distinct local configurations rather than comparable instances of a shared phenomenon. In this sense, multi-country inclusion may generate not only broader relevance, but also greater dependence on contextual interpretation [44].

Overall, this PRO Y&O experience suggests that international research on vulnerability should not assume that a common classificatory framework produces directly comparable populations across countries. Rather, such frameworks may be better understood as heuristic tools that support orientation and coordination, while the actual meaning of vulnerability remains partly context-bound. The challenge, therefore, is not only to adapt interventions to local cultures, but also to make explicit which dimensions of vulnerability are genuinely shared across settings and which remain specific to particular social and institutional environments. Under these conditions, the value of multi-country research may lie less in producing simple generalizations and more in clarifying the limits, conditions, and transferability of vulnerability categories across contexts.

The lesson learned was that a shared conceptual structure can ensure coordination across countries, but analytical comparability requires making explicit which dimensions of vulnerability are common across settings and which are locally specific.

### 3.6. Limitations

Some limitations of this communication paper should be acknowledged.

First, the reflections presented are based on the specific experience of the PRO Y&O project and are therefore contextually situated. The project involved five European countries, a defined consortium structure, and a specific focus on infection-prevention behaviors and educational intervention development. Consequently, the challenges described may not be directly transferable to other research settings, populations, disciplinary fields, or types of intervention.

Second, the reflective material was not generated through a formal qualitative study designed specifically to investigate researchers’ experiences. Rather, it emerged from consortium discussions and decisions documented during project implementation. Although this approach allowed the team to capture challenges as they arose in practice, it also means the material may reflect the issues that were most visible, discussed, or documented by the research team, while other difficulties may have remained implicit or underreported.

Third, reflexivity is inherently influenced by the positionality, disciplinary background, and interpretive perspectives of the researchers involved. Efforts were made to mitigate this limitation through collaborative discussion, review by all consortium members, and oversight by senior researchers. However, the analysis remains shaped by the perspectives of the research team and does not represent an independent external evaluation of the project.

Finally, the multi-country nature of the project increased the richness of the reflections but also introduced heterogeneity in social, cultural, institutional, and healthcare contexts. The same vulnerability category could have different meanings across countries, and the populations selected by each partner were not always analytically equivalent. These insights should therefore be interpreted as contextually grounded contributions to an ongoing methodological and ethical discussion, rather than as generalizable recommendations or a standardized framework for all studies involving people living in situations of vulnerability.

## 4. Conclusions

Discussing vulnerability in research remains essential, not only because some participants may face greater risks of harm, exclusion, or injustice, but also because attention to vulnerability can enhance the quality of relationships established throughout the research process. The experience of the PRO Y&O project suggests that, in international qualitative studies, this attention should be translated into clearly defined methodological choices that ensure personalization while maintaining rigor and sensitivity to context.

Rather than treating vulnerability as a static category assigned to predefined groups, projects involving people living in situations of vulnerability should define it as a working concept: shared enough to ensure coherence across partners, but open enough to reflect local contexts, participant perspectives, and barriers to participation. From this perspective, the main challenge is not merely to classify “who is vulnerable”, but to recognize the elements, circumstances, and contextual conditions that may place individuals at risk, in difficulty, or with reduced access to participation and benefit, and therefore to personalize care and research.

For future multi-country projects, this implies documenting how vulnerability is defined and operationalized in each setting, involving local expertise and, where possible, community perspectives, maintaining reflexive records of ethical and methodological decisions, and clarifying which elements of the intervention require consistency and which can be locally adapted. These commitments may help balance protection with inclusion, comparability with personalization, and ethical consistency with responsiveness to lived realities. At the policy level, this experience suggests that international healthcare and research governance frameworks should not rely only on predefined vulnerability categories or standardized procedural safeguards. Rather, they should support early accessibility planning, linguistic and cultural mediation, documentation of local rationales for involving specific populations, and mechanisms for monitoring ethical and methodological tensions during implementation.

The PRO Y&O experience does not to offer a definitive model for proceeding with vulnerability; rather, it shows how international research can remain methodologically rigorous while being open to contextual variation. Designing studies as adaptive and reflexive processes may support more equitable, respectful, and contextually grounded research practices. In this sense, the lessons learned from PRO Y&O extend the meaning of personalization beyond intervention content alone, emphasizing the need to personalize access, communication, participation procedures, and ethical safeguards according to the lived context of participants and communities. Although these implications should be interpreted as broad orientations rather than prescriptive policy recommendations, they indicate the need for policies that enable contextual adaptation while preserving shared ethical standards across international projects.

## Figures and Tables

**Table 1:** Work packages, leaders and outcomes of the PRO Y&O project.

Work Package (WP)	Leader of the WP	Outcome of the WP
WP1	University of Lublin, Poland	Management and coordination of the project
WP2	University of Udine, Italy	Development of the project’s theoretical framework
WP3	University of Novo Mesto, Slovenia	Development of a tool for assessing behaviors related to infection prevention in the community
WP4	University of Barcelona, Spain	Massive Open Online Course Design
WP5	Karadeniz Technical University, Türkiye	Implementation and dissemination of the Massive Open Online Course

## Data Availability

The original contributions presented in this study are included in the article. Further inquiries can be directed to the corresponding author.

## Abbreviations

The following abbreviations are used in this manuscript:

CDC	Centers for Disease Control and prevention
MOOC	Massive Open Online Course
PRO Y&O	Protecting You & Others
UNESCO	United Nations Educational, Scientific and Cultural Organization
WP	Work Package
